# Risk of myelodysplastic syndrome and acute myeloid leukemia related to PARP inhibitor maintenance line in real-world ovarian cancer patients

**DOI:** 10.1093/oncolo/oyaf243

**Published:** 2025-08-05

**Authors:** Alberto Farolfi, Chiara Casadei, Nicola Gentili, Sara Testoni, Francesca Rusconi, Emilio Francesco Giunta, Nicole Brighi, Giorgia Gurioli, Daniela Montanari, Gema Hernandez Ibarburu, Ugo De Giorgi

**Affiliations:** Medical Oncology, Breast & GYN Unit, IRCCS Istituto Romagnolo per lo Studio dei Tumori (IRST) “Dino Amadori”, Meldola 47014, Italy; Medical Oncology, Breast & GYN Unit, IRCCS Istituto Romagnolo per lo Studio dei Tumori (IRST) “Dino Amadori”, Meldola 47014, Italy; Outcome Research, IRCCS Istituto Romagnolo per lo studio dei Tumori (IRST) “Dino Amadori”, Meldola 47014, Italy; Biostatistics and Clinical Trials Unit, IRCCS Istituto Romagnolo per lo Studio dei Tumori (IRST) “Dino Amadori”, Meldola 47014, Italy; TriNetX, LLC, Cambridge, MA 02140, United States; Department of Medical Oncology, IRCCS Istituto Romagnolo per lo Studio dei Tumori (IRST) “Dino Amadori”, Meldola 47014, Italy; Department of Medical Oncology, IRCCS Istituto Romagnolo per lo Studio dei Tumori (IRST) “Dino Amadori”, Meldola 47014, Italy; Biosciences Laboratory, IRCCS Istituto Romagnolo per lo Studio dei Tumori (IRST) “Dino Amadori”, Meldola 47014, Italy; Medical Oncology, Breast & GYN Unit, IRCCS Istituto Romagnolo per lo Studio dei Tumori (IRST) “Dino Amadori”, Meldola 47014, Italy; TriNetX, LLC, Cambridge, MA 02140, United States; Department of Medical Oncology, IRCCS Istituto Romagnolo per lo Studio dei Tumori (IRST) “Dino Amadori”, Meldola 47014, Italy

**Keywords:** acute myeloid leukemia, myelodysplastic syndrome, ovarian cancer, PARP inhibitor, real-world evidence

## Abstract

**Objective:**

To estimate the risk of myelodysplastic syndrome (MDS) and acute myeloid leukemia (AML) secondary to PARP inhibitors (PARPi), based on the line of treatment, in real-world ovarian cancer (OC) patients.

**Methods:**

Using the TriNetX platform, we compared a cohort (experimental A) of 3402 OC patients treated with first-line maintenance PARPi to a control cohort of 1653 OC patients treated with platinum-based chemotherapy without PARPi. Experimental group B included 356 OC patients treated with PARPi after a platinum-sensitive relapse and was compared to a control cohort of 1503 patients who had not received PARPi after 2 lines of platinum-based chemotherapy. The cohorts were propensity score matched (PSM) 1:1 (experimental A vs control 1 and experimental B vs control 2) for age, race, bevacizumab treatment, and genetic susceptibility to neoplasms. A hazard ratio (HR) was used to compare the incidence of MDS and AML between the matched cohorts.

**Results:**

In the first-line setting, 2 groups of 1346 matched OC patients (mean age 59.8 ± 10.2 SD) were evaluated. The overall incidence of MDS or AML was 1.9% and 0.1% in the experimental A and control groups, respectively (HR = 2.46; 95% CI 1.27-4.75, *P* = .006). For the platinum-­sensitive relapse setting, the HR was 1.76 (95% CI 0.42-7.37, *P* = .432). No significant differences were observed between the various PARPi used.

**Conclusions:**

Our study indicates that PARPi may increase the risk of MDS or AML after first-line maintenance treatment. No significant differences were found across the types of PARPi used.

Implications for practice PARP inhibitors (PARPi) have changed the treatment landscape of ovarian cancer; however, major concerns exist about their induced risk to the development of secondary myeloid neoplasms. Furthermore, the duration of their administration varies between the first-line setting, which lasts 2 to 3 years, and the recurrent setting, which lasts until disease progression. Therefore, we evaluated the risk of PARPi-induced secondary myeloid neoplasms in a real-world population, demonstrating a significant increase for first-line treatment, whereas the increase was not statistically significant in the platinum-sensitive relapse setting. No differences were observed across the range of PARPi used.

## Introduction

Poly-ADP-ribose polymerase (PARP) inhibitors, including olaparib, niraparib, rucaparib, and talazoparib, have shown significant clinical benefits in treating various cancers, such as ovarian, breast, pancreatic, and prostate cancers. However, with their increasing application in clinical care, rare but serious adverse reactions like myelodysplastic syndrome (MDS) or acute myeloid leukemia (AML) have become more common, and consequently, these treatments carry Food and Drug Administration (FDA) warnings.^1^

The standard care for primary or recurrent ovarian cancer includes platinum-based chemotherapy, followed by maintenance therapy to reduce the risk of relapse.^2^ Platinum-based chemotherapy is known to be associated with the risk of developing MDS/AML, and the risk increases with further lines of therapy.^3^^,^^4^ Poly-ADP-ribose polymerase inhibitors are known to be effective as maintenance treatment in ovarian cancer, which has led to major changes in the treatment of this disease, with a gradient in the magnitude of the benefit according to *BRCA1* or *BRCA2* mutations and homologous recombination deficiency (HRD) status. However, the use of PARPi in clinical trials and real-world data has led to warnings of an increased risk of serious adverse hematological events.^5^^,^^6^ A meta-analysis of 18 placebo randomized controlled trials (RCTs) involving 7307 patients revealed that PARP inhibitors significantly increased the risk of MDS and AML compared to the placebo treatments. The incidence of MDS and AML across the PARP inhibitor groups was 0.73%, highlighting the need for further study to improve clinical understanding, especially in the front-line maintenance setting.^7^ Despite these findings, a different meta-analysis of 5739 patients from 14 RCTs showed no significant association between PARP inhibitors and the incidence of MDS and AML, leading to inconsistent conclusions in the current literature.^4^

In ovarian cancer patients, PARP inhibitors have been demonstrated to be effective as maintenance treatment both in first-line treatment^8–11^ and in platinum-sensitive relapse.^5^^,^^6^ Nonetheless, the duration of their administrations varies between the first line, which lasts 2^8^^,^^10^^,^^11^ to 3 years,^9^ and the recurrent setting, which lasts until disease progression or intolerable toxicity. It is currently unclear whether the risk of MDS/AML is linked to the duration of exposure to the drug, previous chemotherapy regimens, or genetic predisposition (eg, *BRCA* mutations).

Our real-world data analysis aims to shed light on the delicate balance between the clinical benefits of PARP inhibitors and their potential risks in developing PARP inhibitor-related MDS and AML, underscoring the importance of vigilant post-marketing surveillance and patient monitoring.^12^

## Methods

### Data source

This is a noninterventional, retrospective study conducted with data obtained from TriNetX, LLC (“TriNetX”). TriNetX is a global federated health research network that provides access to electronic medical records (EMRs) from healthcare organizations (HCOs) worldwide. The analysis was conducted with the TriNetX Global Collaborative Network, which provides access to data containing diagnoses, procedures, medications, laboratory values, and genomic information from approximately 130 million patients from 112 HCOs from around the world. TriNetX data are updated periodically asynchronously and has been used to run multiple studies published in several peer-reviewed scientific journals.

The data collection, processing, and transmission were performed in compliance with the data protection laws of each contributing HCO. This includes the EU Data Protection Law Regulation 2016/679, the General Data Protection Regulation, which regulates the processing of personal data, and the Health Insurance Portability and Accountability Act (HIPAA), the US federal law which protects the privacy and security of healthcare data. The Global Collaborative Networks^13^ is a distributed network, and analytics are performed on anonymized or pseudonymized/de-identified (per HIPAA) data stored at the HCOs, with only aggregate results being returned to the TriNetX platform. Individual personal data does not leave the HCO. TriNetX is ISO 27001:2013 certified and maintains a robust IT security program that protects personal and healthcare data.

### Study population

The patients recruited for the study were selected from the Global Collaborative Network on the TriNetX Platform with over 144 healthcare organizations (HCOs). We formed a group of patients who were diagnosed with malignant ovarian cancer (International Classification of Diseases, Tenth Revision, Clinical Modification [ICD-10-CM]: C56). The first cohort (experimental A) included 3402 patients treated with either carboplatin or cisplatin and then received a PARP inhibitor (olaparib, niraparib, or rucaparib) as their first-line maintenance treatment. The control cohort (control 1) consisted of 1653 ovarian cancer patients (ICD-10-CM: C56), treated with a platinum-based chemotherapy with or without bevacizumab, but had not been treated with a PARP inhibitor.

The second cohort (experimental B) consisted of 356 patients diagnosed with malignant neoplasm of the ovary (ICD-10-CM: C56), who had undergone 2 lines of chemotherapy and were administered a PARP inhibitor as maintenance treatment for their platinum-sensitive relapse. The control 2 cohort consisted of 1503 ovarian cancer patients (ICD-10-CM: C56), who had undergone at least 2 lines of platinum-based chemotherapy but had not been treated with a PARP inhibitor during their treatment journey. For experimental A, 3527 ovarian cancer patients were included.

The primary outcome of our study was evaluating the risk of MDS or AML related to PARP inhibition in the experimental cohorts versus controls. Secondary outcomes were: the median time from the start of PARP inhibitor treatment to the onset of MDS or AML; and the risk of MDS/AML per type of PARP inhibitor (olaparib vs niraparib vs rucaparib). The index date was the first administration of PARP inhibitor for experimental A and experimental B, and the start of the second platinum-based therapy for the control cohort. Cases of MDS that progressed to AML were counted only once.

### Propensity estimation and matching score

Cohorts were propensity-score matched 1:1 (experimental A vs control 1 and subsequently experimental B vs control 2) for age, race, genetic susceptibility to malignant neoplasm of ovary (ICD10CM: Z15.02), neoplasms (ICD10CM: C00-D49), and bevacizumab treatment.

In order to evaluate the role of bevacizumab, we next compare experimental A versus 2 cohorts of OC patients treated with a first-line platinum-based chemotherapy without any maintenance treatment and then with a cohort of patients treated with first-line bevacizumab maintenance, both matched for age, race, genetic susceptibility to malignant neoplasm of ovary (ICD10CM: Z15.02) and neoplasms (ICD10CM: C00-D49).

### Statistical analysis

All analyses were generated with TriNetX platform software (TriNetX) on February 20, 2024. The descriptive statistics of the baseline patient characteristics included absolute value (*n*), the relative incidence (%) for all the categorical variables, and a comparison between cohorts using the standardized mean difference. Continuous variables were presented with mean value and standard deviation and compared with the *t*-test. After propensity score matching, Cox proportional hazard ratios (HRs) were performed to compare the relative risk of developing MDS or AML. The data analysis was limited to 5 years after the index date. Patients were censored after their last record available in TriNetX. The median latency period was calculated in months using the IQR. The range was defined as the interval between the start of treatment with PARP inhibitors and the diagnosis of MDS or AML.

### Role of the funding source

There was no funding source for this study. The data that support the findings of this study are available from TriNetX,  LLC but third-party restrictions apply to the availability of these data. The data were used under license for this study with restrictions that do not allow for the data to be redistributed or made publicly available. However, for accredited researchers, the TriNetX data are available for licensing at TriNetX, LLC. Data access may require a data sharing agreement and may incur data access fees. In accordance with the journal’s guidelines, we will provide our data for independent analysis by a selected team by the Editorial Team for the purposes of additional data analysis or for the reproducibility of this study in other centers if such is requested. All authors had full access to the data in the study and accepted responsibility for the decision to submit for publication.

## Results

### Risk of MDS/AML after first-line maintenance treatment

Before propensity score matching, the study population contained 3402 OC patients and 1653 controls. After propensity score matching, the study population counted 1346 matched pairs, with a mean age of 60.3 years (±9.8 SD). Table 1 lists the covariate differences between the 2 groups before and after matching. Before matching, significant imbalances between the 2 groups were observed concerning age, race, genetic susceptibility to malignant neoplasms, and bevacizumab treatment. Among the 1346 matched pairs, 382 (28.4%) patients in the experimental A, and 541 (40.2%) patients in the control 1 group were treated with pegylated liposomal doxorubicin.

**Table 1.** Ovarian cancer patient characteristics in the first line before and after propensity score matching.

Covariate	Before matching			After matching		
	Treated with PARPi *N *= 3402 (%)	Control *N *= 1653 (%)	*d* ^a^ (%)	Treated with PARPi *N *= 1346 (%)	Control *N *= 1346 (%)	*d* ^a^ (%)
**Age─Mean ± SD (years)**	63.7 ± 11.0	57.0 ± 13.1	0.53	60.3 ± 9.8	60.6 ± 9.7	0.037
**Race**						
**Caucasian**	2334 (68.6%)	935 (57.4%)	0.234	882 (65.5%)	843 (62.6%)	0.060
**Black**	187 (5.5%)	99 (6.1%)	0.025	70 (5.2%)	85 (6.3%)	0.048
**Asian**	262 (7.7%)	231 (14.1%)	0.208	104 (7.7%)	132 (9.8%)	0.074
**Diagnosis**						
**Genetic susceptibility to other malignant neoplasm**	409 (12.0%)	10 (0.6%)	0.482	10 (0.7%)	10 (0.7%)	<0.001
**Medication**						
**Bevacizumab**	1177 (34.6%)	73 (4.5%)	0.821	76 (5.6%)	73 (5.4%)	0.010

a Standard difference.

Abbreviation: PARPi, poly(ADP-ribose) polymerase (PARP) inhibitor.

**Figure 1.**
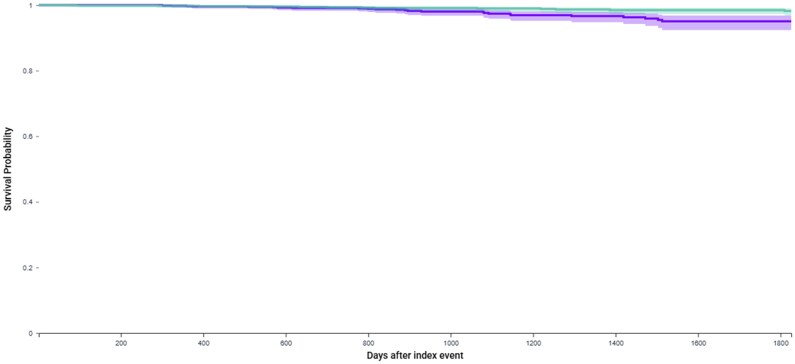
Kaplan-Meier analyses on the risk of myelodysplastic syndrome or acute myeloid leukemia in first line setting.

In the experimental A group, 25 cases of MDS or AML were seen (1.9%), whereas the control 1 group registered 14 (0.1%) cases of MDS or AML. PARP inhibitor therapy significantly increased the risk of MDS or AML vs controls (HR = 2.46; 95% CI 1.27-4.75, *P* = .006), as shown in Figure 1. The median time from the start of a PARP inhibitor to a diagnosis of MDS/AML was 584 days (interval quartile range, IQR 382.5), and from the start of a platinum-based chemotherapy, it was 527 days (IQR 454).

### Risk of PARPi-induced secondary myeloid neoplasms according to bevacizumab treatment

We next compared experimental A with a cohort of patients treated with a platinum-based chemotherapy without maintenance thereafter (neither PARPi nor bevacizumab). We analyzed 1580 matched pairs (mean age 64.8 ± 11.4 SD). Poly-ADP-­ribose polymerase inhibitor therapy significantly increased the risk of secondary myeloid neoplasms versus controls not treated with bevacizumab (HR = 3.42; 95% CI 1.54-7.6, *P* = .001).

When we compared experimental A with a cohort of patients treated with first-line bevacizumab maintenance treatment, we identified 1256 matched pairs (mean age 64.6 ± 11.3 SD). Ovarian cancer patients treated with first-line PARPi maintenance treatment had a risk of HR of 1.05 (95% CI, 0.6-2.0, *P* = .35) of MDS or AML compared to patients treated with first-line bevacizumab.

### Risk of MDS/AML after second-line maintenance treatment

**Figure 2.**
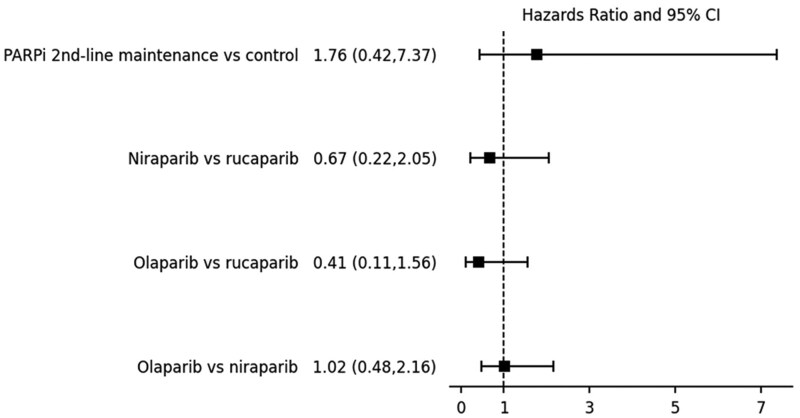
Forrest plot showing the risk of myelodysplastic syndrome or acute myeloid leukemia in platinum-sensitive relapse ovarian cancer.

For the second-line maintenance treatment, the experimental B cohort counted 358 patients before PSM. Propensity score matched generated 263 matched pairs (mean age 60.2 ± 11.2 years), of which 32.3% were treated with bevacizumab (Table 2 lists the covariant differences between the 2 groups before and after matching). Previous treatment with pegylated liposomal doxorubicin was not significantly different between experimental B (31.9% of patients) and the control (36.1%) cohort, HR = 0.91 (95% CI 0.68-1.23, *P* = .544). The incidence of MDS/AML was 5 (1.9%) in experimental B and 3 (1.1%) in the control cohort, respectively (Figure 2): HR = 1.76 (95% CI 0.42-7.37, *P* = .432). The median time to diagnosis of MDS/AML from the start of the second-line maintenance treatment was 206 (IQR 318) and 632 (IQR 151) days from the start of the second-line platinum-based chemotherapy, respectively, in experimental B and control 2 cohort.

**Table 2.** Platinum-sensitive ovarian cancer patient characteristics at first relapse before and after propensity score matching.

Covariate	Before matching			After matching		
	Treated with PARPi *N *= 358 (%)	Control *N *= 1503 (%)	*d* ^a^ (%)	Treated with PARPi *N *= 263 (%)	Control *N *= 263 (%)	*d* ^a^ (%)
**Age─Mean ± SD (years)**	59.9 ± 10.9	57.4 ± 13.3	0.213	60.2 ± 11.2	60.6 ± 10.1	0.039
**Race**						
**White**	259 (72.3%)	836 (59.4%)	0.123	185 (70.3%)	185 (70.3%)	<0.001
**Black**	13 (3.6%)	81 (5.8%)	0.276	10 (3.8%)	10 (3.8%)	<0.001
**Asian**	24 (6.7%)	72 (5.1%)	0.067	18 (6.8%)	16 (6.1%)	0.031
**Diagnosis**						
**Genetic susceptibility to other malignant neoplasm**	76 (21.2%)	10 (0.7%)	0.695	10 (3.8%)	10 (3.8%)	<0.001
**Medication**						
**Bevacizumab**	136 (38.0%)	86 (6.1%)	0.833	85 (32.3%)	85 (32.3%)	<0.001

a Standard difference.

Abbreviation: PARPi, poly(ADP-ribose) polymerase (PARP) inhibitor.

### Risk of MDS/AML according to the type of PARP inhibitor administered

For the last analysis, we compared patients according to the type of PARP inhibitor administered during their treatment journey. Propensity score matching generated 1174 matched pairs (mean age 64.9 ± 10.5 years), of which 30.6% were treated with bevacizumab. 21.1% and 29.5% of patients were treated with pegylated liposomal doxorubicin in the olaparib and niraparib arms, respectively. The incidence of MDS/AML was 14 patients in the olaparib group compared to 13 patients in the niraparib group (HR = 1.02, 95% CI 0.48-2.16; *P* = .979).

Among the 262 matched pairs of patients treated with olaparib and rucaparib (mean age 64.2 ± 10.4 years), 35.5% and 36.6%, respectively, had previously received bevacizumab. Pegylated liposomal doxorubicin was previously used in 23.7% and 29% of patients treated with olaparib and rucaparib, respectively. The incidence of MDS/AML was 5 (1.9%) for olaparib and 3 (1.1%) for rucaparib patients (HR = 0.41, 95% CI 0.11-1.56, *P* = .462).

Finally, propensity score matching generated 262 matched pairs of patients treated with niraparib and rucaparib. The mean age was 64.6 ± 10.4 years. Thirty-six point six percent had previously received bevacizumab, and 24.8% and 29% received pegylated liposomal doxorubicin in each group, respectively. The incidence of MDS/AML was 5 (1.9%) in niraparib and 8 (3.1%) in rucaparib patients (HR = 0.67, 95% CI 0.22-2.05, *P* = .133) (Figure 2).

## Discussion

Poly-ADP-ribose polymerase inhibitors have changed the landscape of ovarian cancer treatment, but some concerns remain about the risk of secondary MDS and AML, rare, but serious, adverse events. Real-world data generating real-world evidence are essential for implementing safety and efficacy profiles in daily clinical practice.^14^ The present study provided data on the risk of developing MDS and AML in a large cohort of real-world ovarian cancer patients who underwent first-line maintenance treatment or platinum-sensitive relapse. As reported in randomized clinical trials,^8–11^ PARP inhibitor therapy has shown statistical significant increase in the risk of MDS or AML, when used as maintenance in ovarian cancer patients as first-line maintenance setting. Reassuringly, the number of events recorded in our real-world experience was low and similar to other findings from randomized control trials and adverse drug reaction registries.^1^^,^^3^^,^^7^

BRCA deficiency has been suggested as a possible risk factor for MDS and AML.^15^ In this context, olaparib, which is mainly used in BRCA-mutated and HRD-positive patients outside of clinical trials, showed the strongest association with MDS and AML compared to other PARP inhibitors. Consequently, we have decided to evaluate the risk of these events in our study by comparing olaparib with other PARP inhibitors. The type of PARP inhibitor used did not show any differences. A meta-analysis of randomized control trials, which investigated different diseases and combinations, including platinum-based regimens and concomitant PARP inhibition, found no differences in terms of the assignment of PARP inhibitors.^7^ This confirmed our findings. Furthermore, no significant difference in the risk of MDS and AML was observed between studies limited to BRCA1/2 mutation carriers and open to all patients.^7^

The clonal selection in hematopoietic stem cells that emerges under the pressure of chemotherapy or other DNA-damaging treatments could ultimately lead to the development of therapy-related myeloid neoplasms.^16^ Patients treated with chemotherapy and exposed to alkylating agents are known to have an increased risk of MDS and AML,^3^ with a median onset of 5.1 years and 3.8 years, respectively.^17^ Most of the mutations identified in secondary MDS and AML belong to the DNA damage response (DDR) pathway, such as PPM1D and TP53, with the latter being common in high-grade serous ovarian carcinoma, occurring in up to 96% of cases.^18^ In gynecological malignancies, about 38% present clonal hematopoiesis with enriched DDR gene mutations.^19^ The risk of developing MDS and AML increases with the duration of exposure to DNA-damaging therapies. It was demonstrated indeed that the prior exposure of platinum compounds or PARPi is associated with an increased risk in a dose-dependent manner for carboplatin for the development of a clonal hematopoiesis resistant to DNA damage-induced apoptosis, providing them with a selective advantage under cytotoxic stress.^20^ In our study, the time to the onset of myeloid-secondary neoplasm was much longer^3^ than the latency we observed in our study with PARP inhibitors. These observations support the hypothesis that PARP inhibitors may act by exerting a selective pressure that stimulates clonal expansion.^21^ Moreover, the loss of statistical significance in the risk of MDS/AML induced by PARP inhibitors between the first-line and second-line settings could be related either to a smaller sample size or to the fact that the control group received a higher number of platinum-based therapy cycles, potentially increasing their risk of developing therapy-related clonal hematopoiesis.

Of note, the median time of onset of MDS or AML from PARPi initiation in our real-world study was about one year and half. The low incidence of this serious adverse event in the first 2 years of treatment, combined with the hypothesis that the lack of benefit on overall survival in the PRIMA study could be related to the occurrence of relapse during treatment with PARPi (in which niraparib was administered for 3 years),^22^ support the idea that perhaps a first-line maintenance therapy should not last more than 2 years. However, this remains a question that needs to be further explored.

The most significant limitation of our study is inherent to the use of real-world data: the identification of patients with MDS or AML relies on diagnostic data recorded according to the International Classification of Diseases, Tenth Revision. However, this analysis is more reliable than reports from pharmacovigilance registries, where reports of adverse events are arbitrary, biased, and with the added risk of underreporting and missing information. This, combined with the large sample size, yields results that are highly consistent, as we were able to account for and address a wide range of potential confounders.

Another limitation of our analysis is the lack of data on clinical relevant information such as the type of surgery (primary debulking vs interval debulking surgery) and eventual presence of residual disease. Moreover, molecular data provided by the Global Network database are very limited. Although the BRCA status was not available in the Global Collaborative Network, we used the family history of neoplasm as an indicator for this event. Furthermore, we used olaparib which, outside of clinical trials, is used exclusively in BRCA-mutated patients and HRD-positive patients (with bevacizumab). We also lack on data about clonal hematopoiesis that might be the result of the pressure of previous treatments, and it should be considered in future prospective studies. Finally, our study lacks data on the individual doses for each patient, as well as a lack of data on subsequent therapies. Therefore, the exact magnitude of our risk estimates, including the proportions of excess cases, should be interpreted cautiously.

In conclusion, our real-world data are reassuring about the use of PARP inhibitors in ovarian cancer patients, having shown that the risk for secondary myeloid neoplasms is low and similar to the one reported in the clinical trials. The median time of the onset of this adverse event in first-line maintenance treatment supports the hypothesis that 2 years should be enough. Furthermore, the previous observation of a higher risk for patients treated with olaparib was not confirmed.

## Acknowledgment

This work was partly supported thanks to the contribution of Ricerca Corrente by the Italian Minestry of Health, whitin the research line «Precision, gender and ethinicity-based medicine and geroscience: genetic-molecular mechanism in the development, characterizaziont and treatment of tumors.

## Author contributions

Alberto Farolfi (Conceptualization, Data curation, Investigation, Methodology, Supervision, Validation, Writing—original draft, Writing—review & editing), Chiara Casadei (Data curation, Writing—review & editing), Nicola Gentili (Funding acquisition, Methodology, Project administration, Software), Sara Testoni (Data curation, Funding acquisition, Software, Writing—original draft), Francesca Rusconi (Data curation, Investigation, Methodology, Writing—original draft), Emilio Francesco Giunta (Investigation, Validation, Visualization), Nicole Brighi (Investigation, Methodology, Validation, Visualization), Giorgia Gurioli (Resources, Visualization, Writing—original draft), Daniela Montanari (Data curation, Visualization, Writing—original draft), Gema Hernandez Ibarburu (Data curation, Formal analysis, Methodology, Software, Writing—original draft, Writing—review & editing), and Ugo De Giorgi (Funding acquisition, Supervision, Validation, Writing—review & editing)

## Data Availability

The data underlying this article were accessed from TriNetX, LLC [www.trinetx.com]. The derived data generated in this research will be shared on reasonable request to the corresponding author.
